# Phenotype-Guided Nanotherapeutic Strategies for Carbapenem-Resistant *Acinetobacter baumannii*: Toward Precision Antimicrobial Intervention

**DOI:** 10.3390/pharmaceutics18060716

**Published:** 2026-06-10

**Authors:** Ayman Elbehiry, Adil Abalkhail, Fahad A. Alhumaydhi, Eman Marzouk

**Affiliations:** 1 Department of Public Health, College of Applied Medical Sciences, Qassim University, P.O. Box 6666, Buraydah 51452, Saudi Arabia; ar.elbehiry@qu.edu.sa (A.E.);; 2 Department of Medical Laboratories, College of Applied Medical Sciences, Qassim University, P.O. Box 6666, Buraydah 51452, Saudi Arabia

**Keywords:** carbapenem-resistant *Acinetobacter baumannii*, phenotypic heterogeneity, antibiotic resistance, nanotherapeutics, adaptive therapy, biofilms, precision medicine

## Abstract

Carbapenem-resistant *Acinetobacter baumannii* (CRAB) is considered a persistent clinical problem characterized by high mortality and restricted therapeutic options. The current antimicrobial regimen focuses on active bacteria without taking into account physiological states that influence the treatment response. Biofilm formation, metabolic changes, efflux activity, and membrane remodeling reduce antibiotic activity at infection sites and help bacteria survive despite in vitro susceptibility. Clinical performance is also compromised by inadequate tissue penetration, toxicity, and inconsistent pharmacokinetics, which reduce the ability to maintain effective antimicrobial activity at the target site. Therefore, a new strategy is needed that considers how bacteria behave during infection. Nanotherapeutic systems can optimize antimicrobial delivery by changing drug distribution and enabling sustained antimicrobial release within infected tissues. These properties can improve antimicrobial distribution within biofilms and structurally restricted infection sites. This review proposes a phenotype-guided approach linking dominant bacterial phenotypes with targeted nanotherapeutic intervention. Advances in nanoscale diagnostics and computational analysis allow earlier identification and more precise characterization of resistance features, so treatment decisions reflect the current state of infection. When integrated with nanotechnology, this information supports treatment approaches that adapt to changes in bacterial behavior over time. Extending this concept to host-directed and microbiome-informed interventions provides additional control by addressing factors that sustain infection beyond the pathogen. These elements create an integrated system that connects detection, analysis, and treatment, allowing therapy to match the biological conditions of infection for more precise CRAB management.

## 1. Introduction

Antimicrobial resistance (AMR) represents a major global health challenge with substantial clinical and socioeconomic impact [1,2]. According to the World Health Organization (WHO), resistant infections are responsible for approximately 700,000 deaths annually worldwide, with projections estimating up to 10 million deaths per year by 2050 if effective interventions are not developed [3,4,5]. In response to this threat, the WHO identified priority bacterial pathogens because of resistance to conventional antibiotics and their association with prolonged hospitalization, increased healthcare costs, and higher mortality [6,7].

Among these pathogens, *Acinetobacter baumannii* (*A*. *baumannii*) has emerged as a major cause of healthcare-associated infections due to its rapid evolution into a multidrug-resistant (MDR) organism [8]. Carbapenem-resistant *A. baumannii* (CRAB) is particularly problematic in hospital settings, where it contributes to ventilator-associated pneumonia (VAP), bloodstream infections, wound infections, and other severe infections associated with high morbidity and mortality in critically ill patients [9,10]. In CRAB-associated respiratory infections, interactions between inhaled nanotherapeutic systems and pulmonary surfactants may additionally affect local antimicrobial distribution and nanoparticle (NP) behavior within the lung microenvironment [11,12].

Although genetic resistance mechanisms contribute substantially to CRAB survival, treatment failure cannot be explained solely by acquired resistance genes. Experimental and clinical observations demonstrate that phenotypic variability strongly influences antimicrobial response under infection conditions that differ from standardized laboratory testing [13,14]. CRAB combines multiple resistance-associated mechanisms, including enzymatic antibiotic inactivation, reduced outer membrane permeability, and active efflux, all of which limit intracellular antimicrobial accumulation [15,16]. In parallel, phenotypic states such as biofilm formation, metabolic adaptation, and transient persistence further reduce antimicrobial effectiveness by restricting drug penetration, altering physiological activity, and promoting survival during exposure [13,17,18].

Over the past decade, carbapenem resistance in *A. baumannii* has increased steadily worldwide, with several endemic regions reporting resistance rates exceeding 90% [19]. Consequently, treatment strategies increasingly rely on last-resort agents associated with limited efficacy and considerable toxicity [9]. Current antimicrobial approaches remain largely empirical and are primarily guided by susceptibility testing performed under standardized laboratory conditions [20]. However, these conditions do not adequately reflect the heterogeneous microenvironments encountered during infection, where bacterial populations may exist in slow-growing, dormant, or biofilm-associated states with variable antimicrobial susceptibility [13,17,21]. This discrepancy contributes to inconsistencies between in vitro susceptibility profiles and clinical treatment outcomes.

At the same time, antimicrobial development has not kept pace with the emergence of resistance. Many newly introduced agents remain vulnerable to pre-existing or rapidly acquired resistance mechanisms, highlighting the limitations of approaches based primarily on incremental modification of existing antibiotics [20,22,23].

Nanotechnology provides an alternative strategy for improving antimicrobial delivery and activity within complex infection environments. Nanomaterial-based systems can modify drug distribution, facilitate interaction with bacterial structures, and increase antimicrobial activity within restrictive infection environments [24,25,26,27]. Some nanomaterials additionally exhibit intrinsic antimicrobial properties, thereby expanding their therapeutic potential [28]. Nevertheless, optimized delivery alone is insufficient to address all determinants of treatment failure because therapeutic capability remains strongly influenced by bacterial physiological state and adaptive variability [21,29].

In this context, this review proposes a phenotype-guided nanotherapeutic framework for CRAB management that links dominant phenotypic constraints with corresponding nanotherapeutic strategies. Rather than focusing solely on descriptive resistance mechanisms, this framework emphasizes the functional determinants governing antimicrobial failure and aligns therapeutic design with the biological conditions limiting antimicrobial activity during infection. The following sections examine major phenotypic constraints, evaluate pharmacological limitations of current therapies, and discuss how nanotherapeutic platforms may support mechanism-informed intervention strategies in CRAB infections.

Several clinically approved nanotherapeutic formulations have already demonstrated the clinical potential of nanotechnology-based drug delivery. Liposomal formulations such as liposomal amikacin and liposomal amphotericin B have shown improved drug stability and targeted tissue distribution, and reduced systemic toxicity in clinical use [12,30]. Although these systems were not specifically developed for CRAB infections, they provide important translational evidence supporting the feasibility of nanomaterial-assisted antimicrobial therapy. These advances support continued development of phenotype-guided nanotherapeutic strategies against MDR Gram-negative pathogens.

**Figure 1:**
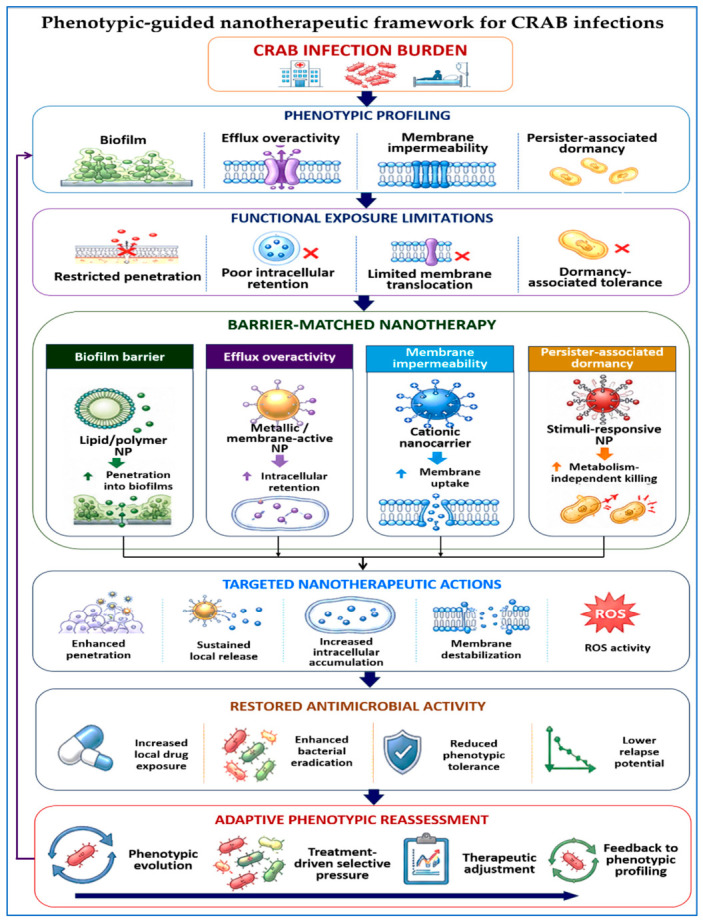
Phenotype-guided nanotherapeutic framework for CRAB infections. Phenotypic profiling identifies major barriers, including biofilm formation, efflux overactivity, membrane impermeability, and persister-associated dormancy. These barriers create distinct limitations in antimicrobial exposure and guide the selection of barrier-matched nanotherapeutic systems. Targeted nanotherapy improves drug penetration, intracellular retention, and antibacterial activity, resulting in enhanced bacterial eradication and reduced relapse potential. Continuous phenotypic reassessment supports adaptive therapeutic adjustment.

Figure 1 illustrates a phenotype-guided nanotherapeutic framework for CRAB infections, linking dominant phenotypic barriers with barrier-matched nanotherapeutic strategies and adaptive therapeutic refinement.

## 2. Phenotypic Determinants of Resistance and Persistence

CRAB exhibits multiple adaptive characteristics that influence antimicrobial effectiveness by altering drug entry, intracellular retention, and bacterial physiological activity [31,32]. These mechanisms collectively reduce intracellular antibiotic exposure [33,34]. Permeability restriction, efflux activity, biofilm growth, and metabolic adaptation act together to promote persistence during treatment [16,35,36,37].

### 2.1. Biofilm-Associated Structural Complexity

Surface-associated growth enables *A. baumannii* to persist on host tissues and medical devices, including ventilatory equipment [38,39]. Within biofilms, bacterial cells are embedded in an extracellular matrix composed of polysaccharides, proteins, and extracellular DNA that limits exposure to environmental stress and antimicrobial agents [40].

This matrix restricts antimicrobial diffusion, generating concentration gradients that reduce drug availability within deeper biofilm layers [17,40]. Simultaneously, oxygen and nutrient gradients create heterogeneous microenvironments containing bacterial populations with variable physiological activity [40]. Cells located in nutrient-deprived regions frequently exhibit reduced metabolic activity, limiting antibiotics that depend on active cellular processes.

Biofilm-associated populations can tolerate antibiotic concentrations substantially higher than those affecting planktonic cells [39]. Quantitative susceptibility studies further demonstrate marked differences between planktonic and biofilm-associated *A. baumannii* populations. Reported biofilm eradication concentrations (MBECs) for cefotaxime, imipenem, and ciprofloxacin were substantially higher than corresponding planktonic MIC values, with increases ranging 8–2048-fold, 32–512-fold, and 16–512-fold, respectively [41]. These findings highlight the partial efficacy of conventional antimicrobial exposure against established biofilm-associated infections.

In addition, biofilms contain dormant subpopulations capable of surviving antimicrobial exposure without stable genetic resistance [42,43]. The combined effects of restricted penetration, physiological heterogeneity, and metabolically inactive subpopulations reduce bacterial eradication and support persistence after treatment [17,44].

Quorum sensing (QS) also contributes to CRAB persistence through regulation of biofilm maturation, surface colonization, and bacterial stress adaptation. In *A. baumannii*, the AbaI/AbaR signaling system has been associated with acyl-homoserine lactone-mediated communication involved in biofilm development, and disruption of this pathway may impair later stages of biofilm maturation [45,46]. Some nanotherapeutic systems have additionally been explored as potential modulators of bacterial communication networks through effects on signaling pathways, membrane interactions, and biofilm-associated physiological activity [47]. However, the precise contribution of QS to CRAB persistence remains incompletely understood, and the clinical relevance of QS-targeted nanotherapeutic approaches requires further investigation.

### 2.2. Efflux-Mediated Drug Extrusion

Active efflux systems play a major role in limiting intracellular antibiotic accumulation. In *A. baumannii*, resistance-nodulation-division (RND) transporters, particularly AdeABC, contribute substantially to multidrug resistance through extrusion of structurally diverse antimicrobial agents, including β-lactams, aminoglycosides, tetracyclines, and fluoroquinolones [34,48,49].

These transporters function through proton motive force-dependent mechanisms and form tripartite complexes spanning the bacterial envelope, enabling direct extrusion of antimicrobial compounds into the extracellular space [33,50]. As a result, intracellular drug levels may become insufficient for effective target engagement [34,51].

Because RND efflux systems depend on proton motive force-driven transport [33], disruption of bacterial membrane energetics may impair efflux activity and increase intracellular antimicrobial accumulation. Some NP systems have been reported to disrupt membrane integrity and induce oxidative stress-related metabolic disturbances [52,53]. These effects may reduce drug extrusion and improve intracellular retention. Nevertheless, the direct role of NP-mediated energy disruption in efflux inhibition during CRAB infection remains unclear.

Regulatory systems such as AdeRS further modulate efflux activity in response to environmental stress and antimicrobial exposure [51,54]. Beyond direct drug extrusion, efflux-associated systems also contribute to bacterial survival under stress through effects on bacterial signaling and adaptive regulatory pathways that support persistence during antimicrobial exposure [34,50,55].

### 2.3. Outer Membrane Remodeling and Reduced Permeability

The outer membrane of *A. baumannii* acts as a selective permeability barrier that restricts antibiotic entry, particularly for hydrophilic agents dependent on porin-mediated transport [16,56]. Reduced influx limits intracellular drug accumulation and reduce antimicrobial activity [56].

Alterations in outer membrane proteins further strengthen this barrier. The carbapenem-associated porin CarO contributes to imipenem uptake, and its loss or structural modification has been associated with reduced permeability and increased resistance in clinical isolates [31,57,58,59]. Additional porins, including proteins within the 33–36 kDa range, also influence permeability regulation [31].

Unlike many Gram-negative bacteria, *A. baumannii* lacks large nonspecific porins such as OmpF and OmpC, resulting in intrinsically reduced membrane permeability even in the absence of acquired resistance mechanisms [16]. Changes in membrane lipid composition may further reduce antibiotic penetration [56].

Reduced permeability frequently acts synergistically with efflux activity. Limited influx decreases intracellular entry, while active extrusion removes compounds that successfully cross the membrane, together minimizing intracellular antimicrobial exposure [31,34,56].

### 2.4. Persister Cell Formation and Transient Tolerance

A distinct subpopulation of bacterial cells can survive antimicrobial exposure through a reversible tolerant physiological state [13]. Because many bactericidal antibiotics require active metabolic or biosynthetic processes, reduced cellular activity decreases antimicrobial susceptibility despite adequate drug exposure [60]. Following removal of treatment pressure, these cells may resume growth and contribute to recurrent infection [44].

Environmental stress conditions, including nutrient limitation, oxidative stress, and antimicrobial exposure, increase the formation of persister populations, particularly within structured bacterial communities such as biofilms [17,60]. Transitions into this state are associated with global metabolic downregulation and activation of stress-response pathways, including toxin–antitoxin systems that facilitate switching between active growth and dormancy [61].

Clinically, persister-associated survival contributes to delayed bacterial clearance, chronic infection, and relapse despite apparent susceptibility in laboratory testing, which primarily reflects responses of actively dividing bacterial populations [44]. The major phenotypic determinants influencing antimicrobial performance in CRAB, their clinical implications, and representative nanotherapeutic intervention strategies are summarized in Table 1.

## 3. Pharmacological Limitations of Current Antimicrobial Therapies

Current antimicrobial regimens remain inadequate against CRAB because therapeutic success is frequently limited by insufficient drug exposure at the site of infection [62,63]. Clinical response may remain poor despite documented susceptibility because standardized testing does not capture pharmacokinetic (PK) variability or phenotype-associated survival mechanisms [63].

### 3.1. Toxicity Constraints of Last-Resort Agents

Polymyxins, including colistin and polymyxin B, are commonly used in CRAB management but are associated with substantial toxicity that restricts their clinical utility [63]. Nephrotoxicity is the most frequent adverse effect, with reported incidence ranging from 30% to 60% depending on patient characteristics and dosing strategies [64].

This toxicity profile limits dose escalation because attempts to increase antibacterial exposure simultaneously increase the risk of renal injury [65]. Consequently, polymyxins exhibit a narrow therapeutic window in which insufficient exposure compromises efficacy while higher exposure increases toxicity risk [65].

Pharmacodynamic (PD) performance may also remain inadequate despite aggressive dosing. Experimental pulmonary infection models demonstrate that polymyxins may fail to maintain sustained bacterial suppression even near tolerated exposure limits [66]. These limitations reduce the reliability of polymyxins in severe CRAB infections.

### 3.2. Inadequate Pharmacokinetics and Tissue Penetration

Successful antimicrobial therapy depends on achieving sufficient drug concentrations at the site of infection; however, many agents used against CRAB demonstrate highly variable PK behavior [62]. In critically ill patients, altered organ function, fluid shifts, and changes in distribution volume contribute to unpredictable antimicrobial exposure and increase the risk of subtherapeutic concentrations [63].

Pulmonary infections represent a particular challenge because polymyxins exhibit inconsistent penetration into lung compartments, reducing therapeutic efficiency in respiratory disease [66]. Interactions between NPs and pulmonary surfactants can influence particle stability, antimicrobial distribution, and local drug delivery within the respiratory tract [12,67]. Surfactant adsorption can modify NP surface properties and affect retention and diffusion within infected lung tissues, particularly in VAP [11,68]. These factors should therefore be considered during development of inhaled nanotherapeutic strategies for CRAB respiratory infections.

Protein binding and tissue compartmentalization can further decrease the proportion of pharmacologically active drug within infected tissues, particularly in deep-seated or structurally complex infections [62]. As a result, antimicrobial concentrations sufficient for bacterial eradication may not be achieved despite apparently appropriate dosing regimens [62,63].

Emerging PK/PD modeling approaches can support the development of nanotherapeutic strategies against CRAB infections by integrating NP distribution, drug-release kinetics, tissue penetration, and antimicrobial exposure over time [65,69]. These approaches may better characterize antimicrobial behavior in biofilms and poorly perfused tissues where plasma PK parameters do not accurately reflect tissue-level exposure. PK/PD evidence also suggests that antimicrobial exposure-response relationships can vary according to infection site and tissue penetration characteristics [69]. Nonetheless, PK/PD frameworks for nano-antimicrobials remain underdeveloped and require further experimental and clinical validation [65,69].

### 3.3. Heteroresistance and Phenotypic Survival

Heteroresistance represents an additional source of variability in antimicrobial response and is characterized by the presence of bacterial subpopulations with reduced susceptibility within a single isolate [70]. This phenomenon has been widely reported in *A. baumannii*, particularly during polymyxin exposure, where resistant subpopulations may expand under treatment pressure [71].

Reported prevalence varies substantially between studies because detection depends heavily on methodology and population characteristics [72,73,74]. Nevertheless, even low-frequency resistant subpopulations can contribute to incomplete bacterial eradication by surviving antimicrobial exposure and subsequently re-establishing growth [70].

Because these subpopulations may remain below routine detection thresholds, standard susceptibility testing can underestimate clinically relevant resistance, contributing to discrepancies between laboratory susceptibility profiles and therapeutic response [70,71].

Stress-responsive regulatory pathways further contribute to survival during antimicrobial exposure. Regulatory systems such as PhoPQ and PmrAB alter membrane composition and modulate susceptibility in response to environmental stress [75]. These reversible responses increase phenotypic heterogeneity and reduce treatment predictability under clinical conditions [72,75].

### 3.4. Absence of Phenotype-Oriented Drug Design

Most currently available antimicrobial agents were developed to target essential processes in actively dividing bacteria, including cell wall synthesis, protein production, and nucleic acid replication [76]. Although effective under standardized laboratory conditions, these approaches do not adequately address the functional states encountered during infection.

Experimental evidence demonstrates that *A. baumannii* can undergo phenotypic changes involving altered permeability, active efflux, metabolic adjustment, and transient tolerance that reduce antimicrobial efficacy without directly changing intrinsic drug potency [77]. Conventional antimicrobial development has largely focused on modification of existing antibiotic classes rather than direct targeting of these functional resistance-associated states [78].

Consequently, major barriers including restricted intracellular exposure, biofilm-associated protection, and dynamic physiological adaptation remain insufficiently addressed by current therapeutic strategies. Persistent CRAB infection therefore reflects a mismatch between antimicrobial mechanism and bacterial physiological state during infection [13,37]. These limitations support the need for phenotype-oriented therapeutic approaches in which antimicrobial delivery and activity are aligned with the biological conditions governing resistance and persistence.

Other emerging antimicrobial approaches have also been investigated for CRAB management, including bacteriophage therapy, antimicrobial peptides, CRISPR-based systems, and combination antibacterial regimens [79,80]. These strategies may provide advantages such as strain-specific targeting, membrane-disruptive activity, or direct modulation of resistance-associated genes. However, many remain limited by challenges related to stability, delivery, resistance development, or clinical standardization [81,82]. In this context, phenotype-guided nanotherapeutic strategies may offer complementary advantages by improving antimicrobial distribution, intracellular retention, and localized activity within heterogeneous infection environments.

## 4. Nanotherapeutic Platforms for Antimicrobial Delivery

Nanotechnology modifies antimicrobial distribution and influences drug exposure within infected tissues. Beyond drug delivery, nanotherapeutic systems may promote bacterial interaction, tissue penetration, and local antimicrobial activity [83]. These properties are particularly relevant when conventional formulations fail to maintain effective antimicrobial concentrations at infection sites [84].

### 4.1. Lipid-Based Nanocarriers

Lipid-based nanocarriers, including liposomes, facilitate antimicrobial delivery through interaction with bacterial and host lipid membranes, promoting fusion and intracellular transport of encapsulated compounds [85,86]. Their structural compatibility with biological membranes improves transfer across lipid barriers and facilitates access to intracellular targets poorly reached by free-drug formulations.

Surface charge affects electrostatic interaction with negatively charged bacterial outer membranes, where cationic formulations increase membrane association and antimicrobial uptake [87]. Surface chemistry and hydrophobicity may further influence membrane insertion and diffusion across lipid-rich environments [88], while NP size and shape can affect biofilm penetration and interaction with bacterial surfaces [89,90].

In addition to improving delivery, lipid-based systems increase physicochemical stability by protecting labile antimicrobial agents from degradation [89]. However, exposure to physiological ionic environments and serum proteins may alter NP stability and surface behavior after systemic administration [69,91]. Experimental studies demonstrate enhanced antibacterial activity of liposomal formulations against resistant *A. baumannii*, particularly within biofilm-associated environments where diffusion is restricted [90,92].

Preclinical investigations further show that lipid-based nanoformulations can reduce bacterial burden and increase survival in infection models [92]. Lipid NP systems loaded with lipophilic adjuvants have also been reported to potentiate colistin activity against *A. baumannii* in experimental models such as *Galleria mellonella* [93].

Mechanistically, liposomal systems may enhance interaction between polymyxins and bacterial membranes by increasing binding to lipid A and promoting outer membrane destabilization [68]. Targeted liposomal formulations can additionally improve localized delivery efficiency and facilitate intracellular antimicrobial accumulation [94].

Quantitative analyses indicate that liposomal nanoformulations may reduce minimum inhibitory concentrations (MICs) by approximately 2–4-fold in CRAB isolates [95]. Prolonged retention within infected tissues can enhance antimicrobial exposure, particularly in pulmonary infections with limited conventional drug penetration [96].

### 4.2. Polymeric NPs

Polymeric NPs support sustained antimicrobial release, maintaining therapeutic concentrations over extended periods while reducing fluctuations associated with conventional dosing [83]. Encapsulation within polymeric matrices additionally protects antimicrobial compounds from enzymatic degradation and environmental inactivation, thereby prolonging antimicrobial stability [83].

Surface engineering further influences NP interaction with bacterial envelopes and infected tissues, promoting adherence, uptake, and penetration within structured microbial communities. In *A. baumannii*, chitosan-based NPs have demonstrated membrane-disruptive activity and strengthened bactericidal effects relative to free-drug formulations [90].

Additional studies show that peptide-loaded chitosan NPs can increase membrane permeability, inhibit biofilm formation, and disrupt established biofilms [97]. Biodegradable polymeric systems such as PLGA have similarly demonstrated improved bacterial clearance in experimental infection models through sustained delivery and controlled antimicrobial exposure [98].

Some polymeric nanoplatforms may also modulate host immune responses and increase survival in preclinical infection models [99]. Moreover, photoactive polymeric nanostructures combine controlled delivery with photodynamic antimicrobial activity, providing bactericidal mechanisms that extend beyond conventional antibiotic targets [100].

### 4.3. Metallic NPs

Metallic NPs exhibit intrinsic antimicrobial activity independent of conventional antibiotic mechanisms. In *A. baumannii*, these effects are associated with oxidative stress generation, membrane destabilization, disruption of cellular structures, and impairment of essential biological processes [52,53].

Silver NPs (AgNPs) have been shown to increase membrane permeability and induce leakage of intracellular contents, resulting in loss of bacterial membrane integrity [53]. Concurrent oxidative stress further contributes to protein dysfunction and broader cellular damage [52]. Because these mechanisms are not entirely dependent on bacterial replication, metallic NPs may retain activity across different physiological states [52,53].

This multi-target mode of action supports activity against MDR isolates, including strains resistant to carbapenems and polymyxins [101]. Combination approaches further demonstrate that metallic NPs can enhance antibiotic efficacy by facilitating drug entry and intracellular accumulation. Synergistic activity has been reported for combinations of AgNPs with agents such as colistin and imipenem against resistant *A. baumannii* isolates [102,103].

Rapid membrane disruption caused by metallic NPs may also increase endotoxin release from Gram-negative bacteria during bacterial killing [52,53]. In CRAB infections, excessive lipopolysaccharide release intensifies inflammatory responses, particularly during severe infection [33]. Although this effect remains insufficiently characterized in nano-antimicrobial systems, it highlights the importance of balancing rapid antibacterial activity with potential host inflammatory effects.

Compared with conventional antibiotics that usually target specific bacterial pathways, metallic NPs act through multiple mechanisms, including membrane damage, oxidative stress, and disruption of cellular structures [52,53,101]. This broader activity may reduce the risk of rapid resistance development. However, bacteria can still develop adaptive responses to metallic NPs, including membrane changes, oxidative stress defense mechanisms, and biofilm-associated tolerance [104,105]. Therefore, although metallic NPs may help overcome some conventional resistance mechanisms, their long-term effects on resistance development remain unclear and require further investigation.

### 4.4. Stimuli-Responsive Nanoplatforms

Stimuli-responsive nanoplatforms enable controlled antimicrobial release in response to environmental conditions associated with infection, including pH variation, oxidative stress, and enzymatic activity [83]. These systems aim to localize antimicrobial activation while limiting systemic exposure.

pH-responsive systems have been widely investigated because acidic microenvironments commonly develop within infected tissues and biofilms. Under these conditions, pH-sensitive nanoplatforms can increase localized antimicrobial release in Gram-negative infections, including *A. baumannii* [106,107].

Reactive oxygen species (ROS)-responsive systems provide an additional strategy in which elevated oxidative stress triggers antimicrobial release. Experimental studies suggest that ROS-mediated activation may increase antibacterial activity while reducing off-target exposure [24,25].

Enzyme-responsive systems similarly exploit bacterial enzymatic activity to initiate localized drug release. Although direct evidence in *A. baumannii* remains limited, findings from related Gram-negative models support the potential applicability of this approach [24].

Localized drug release within biofilm-associated regions can improve antimicrobial exposure in structurally restricted infection sites [24,25]. However, biofilm heterogeneity, extracellular matrix barriers, and persister-cell survival continue to limit complete antimicrobial eradication and efficient NP penetration [108,109,110,111,112].

**Figure 2:**
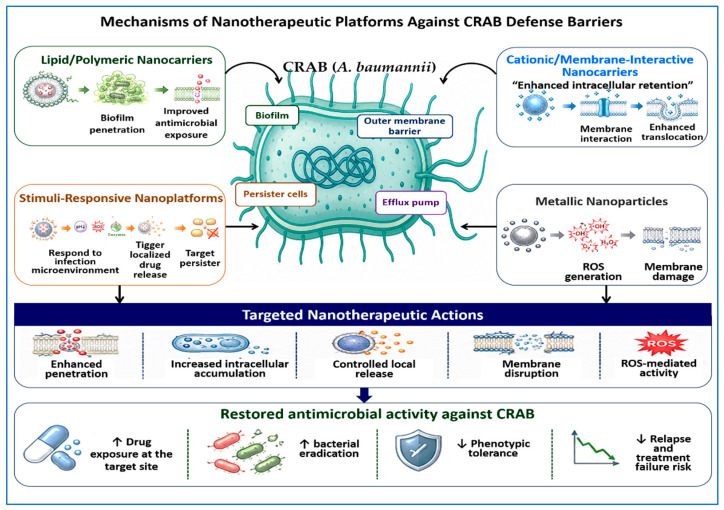
Mechanisms by which nanotherapeutic platforms overcome major CRAB defense barriers. Different nanoplatforms enhance biofilm penetration, intracellular accumulation, localized drug release, membrane disruption, and ROS-mediated antibacterial activity, leading to improved antimicrobial efficacy against CRAB.

Evidence evaluating responsive nanoplatforms in *A. baumannii* is still sparse, and further validation is required before therapeutic implementation [24]. Table 2 summarizes the principal advantages, limitations, and representative antimicrobial characteristics of the major nanotherapeutic platforms investigated against CRAB. Figure 2 illustrates how different nanotherapeutic platforms overcome key CRAB defense barriers and restore antimicrobial activity.

## 5. Phenotype-Guided Precision Nanotherapeutics

Conventional antimicrobial strategies do not adequately account for the phenotypic states governing bacterial survival during infection. In CRAB, treatment outcome is strongly influenced by variability affecting drug entry, intracellular retention, stress responses, and antimicrobial distribution within infected tissues [21,29].

Treatment failure often reflects insufficient intracellular drug exposure caused by limited penetration, retention, or target access [115,116,117,118,119]. Different phenotypic states disrupt these processes through distinct mechanisms, creating dominant functional barriers that limit antimicrobial activity.

This review proposes a phenotype-guided framework linking dominant phenotypic states with corrective nanotherapeutic strategies through identification of the primary exposure limitation and selection of an appropriate delivery system. Within this model, biofilm formation, efflux activity, membrane impermeability, and persister-associated tolerance represent major determinants of therapeutic failure in CRAB [21,29].

**Figure 3:**
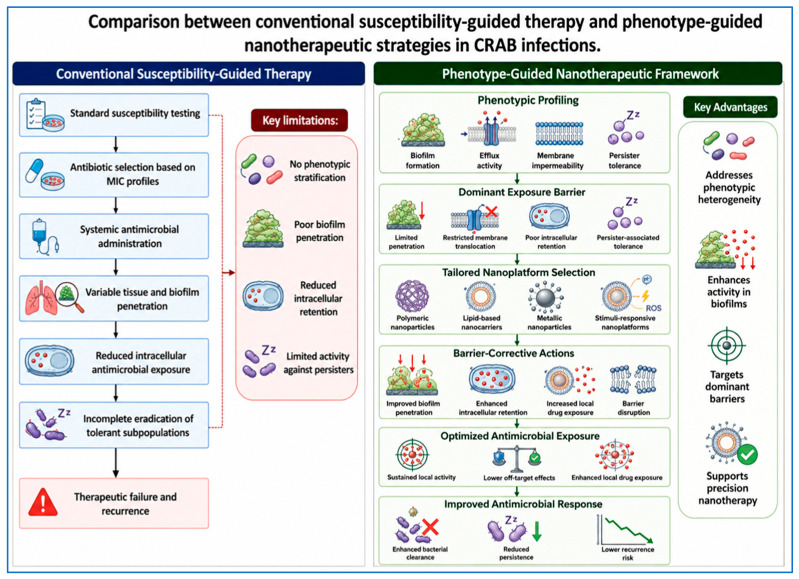
Comparison between conventional susceptibility-guided therapy and a phenotype-guided nanotherapeutic framework for CRAB infections. Phenotypic profiling enables barrier-directed nanoplatform selection to improve antimicrobial exposure, reduce persistence, and enhance treatment response.

These constraints often overlap and vary with infection stage and site [118,119]. Consequently, the framework should be considered a structured decision-support model rather than a deterministic therapeutic algorithm. Figure 3 presents a conceptual comparison between conventional susceptibility-guided therapy and the proposed phenotype-guided nanotherapeutic framework in CRAB infections.

### 5.1. Targeting Biofilm-Driven Structural Barriers

Biofilm formation in *A. baumannii* creates highly organized bacterial communities that reduce susceptibility to antimicrobial agents and host defenses, contributing to persistent infection [120]. Spatial heterogeneity within biofilms generates gradients in nutrient availability and antimicrobial penetration, exposing deeper bacterial populations to sublethal drug concentrations despite adequate external levels [52,108,109].

Quantitative differences between planktonic and biofilm-associated susceptibility reflect this barrier, with biofilm-associated MIC, MBC, and MBEC values frequently exceeding those observed in planktonic populations [41,42,121].

NP-based systems may partially overcome these limitations through interaction with extracellular matrix components and improved intrabiofilm distribution. AgNPs conjugated with chitosan have been reported to reduce MICs and disrupt biofilm formation more effectively than individual components in carbapenem-resistant isolates [122]. Chitosan-based nanostructures have also demonstrated substantial reductions (96–99%) in biofilm biomass under experimental conditions [123].

Additional mechanisms include disruption of biofilm-associated gene regulation, extracellular matrix destabilization, membrane damage, and intracellular leakage [124,125].

However, therapeutic efficacy remains dependent on sufficient penetration depth. Dense matrix composition, polymicrobial organization, and heterogeneous biofilm architecture may restrict NP diffusion and permit survival of protected bacterial subpopulations despite surface disruption [17,40,110].

### 5.2. Overcoming Efflux-Driven Drug Extrusion

Efflux systems reduce intracellular antibiotic accumulation through active export of antimicrobial agents [126,127]. RND transporters, particularly AdeABC, contribute substantially to multidrug resistance in *A. baumannii* by removing structurally diverse compounds from periplasmic and cytoplasmic compartments [48,128].

Antimicrobial entry may occur, but intracellular retention remains insufficient for sustained target engagement. Accurate therapy therefore requires intracellular accumulation that exceeds export capacity [33,50,129].

Nanomaterial-based strategies may interfere with efflux-associated resistance through several mechanisms [130]. Metallic NPs can disrupt membrane-associated transport activity, whereas chitosan-containing nanocomposites have been reported to downregulate efflux-associated genes such as *adeB* [131,132]. Some NP systems may additionally disrupt proton motive force-dependent membrane energetics, thereby impairing energy-dependent efflux function [105,133].

Combination approaches integrating NPs with conventional antimicrobials may further enhance intracellular drug retention and antimicrobial efficacy [134,135]. Nevertheless, dynamic regulatory responses and variable in vivo conditions may reduce the durability of these effects during clinical infection [48,54].

### 5.3. Addressing Permeability-Restricted Entry

Reduced outer membrane permeability limits antibiotic uptake in *A. baumannii*, decreasing intracellular drug availability and compromising antimicrobial activity [136]. This mechanism is particularly relevant in isolates with altered or reduced porin expression, where restricted entry rather than intrinsic resistance becomes the dominant limitation [56,136].

Nanotherapeutic systems can partially overcome this barrier through direct interaction with the bacterial envelope and promotion of alternative transport pathways that partially bypass porin-dependent uptake [137,138]. NP physicochemical properties, including surface charge, hydrophobicity, and morphology, strongly influence membrane interaction and translocation efficiency [138,139].

Effective intervention requires balanced membrane perturbation. Insufficient interaction may fail to improve intracellular delivery, whereas excessive membrane disruption may increase toxicity through oxidative stress, inflammatory responses, and unintended host–cell interactions [87,88,140,141]. Variability in membrane composition between isolates may further influence therapeutic consistency [72,76].

Excessive membrane disruption and oxidative stress can trigger transient bacterial adaptation pathways linked to phenotypic plasticity and stress tolerance. Recent studies show that ROS-mediated and photodynamic stress can induce reversible changes such as metabolic shifts, biofilm-associated heterogeneity, and small-colony variant formation rather than stable resistance [118]. Inorganic NPs may further intensify these effects through multi-target damage to bacterial membranes and intracellular components [47].

Clinical translation of multifunctional nanoplatforms may also be restricted by challenges related to large-scale manufacturing, batch reproducibility, formulation stability, and consistent physicochemical characterization [142,143,144,145]. These factors may affect therapeutic reliability and complicate industrial production and regulatory standardization of complex nanotherapeutic systems.

### 5.4. Eliminating Persister-Associated Survival

Persister cells survive antimicrobial exposure through transient states of reduced metabolic activity without acquiring stable genetic resistance [43]. Under these conditions, intracellular antimicrobial accumulation may not result in effective bacterial killing because of reduced metabolic and target activity [146].

Strategies targeting persisters therefore rely on mechanisms that remain active independent of bacterial replication. Antimicrobial peptides may eliminate persisters through direct membrane disruption [147], whereas metallic nanomaterials can induce oxidative injury and membrane damage even in metabolically inactive cells [105]. Ferroptosis-like bacterial death associated with metal-dependent oxidative stress has also been proposed as a potential contributor to NP-mediated killing, although its relevance in CRAB requires more clarification [148,149].

Sustained antimicrobial exposure may further improve eradication during transitions between dormant and metabolically active states [43,150]. However, prolonged oxidative or membrane-directed activity may increase the risk of host toxicity at higher effective concentrations [140,141,151].

### 5.5. Integrated Phenotype-Oriented Therapeutic Design

CRAB infections commonly involve the coexistence of multiple phenotypic constraints acting simultaneously [38]. The relative importance of these constraints varies across infection stages and anatomical sites [118,119].

Multifunctional nanotherapeutic systems attempt to address this complexity through coordinated effects on membrane interaction, intracellular retention, and antimicrobial distribution [130,152]. Combination strategies integrating nanomaterials with conventional antibiotics can enhance penetration, persistence, and local drug exposure through complementary mechanisms [153,154].

Recent advances in phage-based therapeutics have also introduced engineered delivery systems and CRISPR-assisted platforms to promote activity against MDR pathogens. Encapsulation approaches including hydrogels, liposomes, NPs, and biopolymeric carriers may enhance phage stability, controlled release, biofilm penetration, and localized delivery [79]. Moreover, phage-mediated CRISPR-Cas systems enable targeted disruption of resistance-associated genes while improving antibacterial specificity [81]. Hybrid NP and liposome-based CRISPR delivery systems have further demonstrated improved intracellular targeting and therapeutic stability in biofilm-associated infections [81]. Table 3 summarizes major phenotypic constraints, corresponding nanotherapeutic strategies, and their functional impact on antimicrobial performance.

### 5.6. Clinical Translation of Phenotype-Guided Nanotherapy

Clinical application of phenotype-guided nanotherapy remains limited by the difficulty of directly characterizing phenotypic constraints during infection [155,156,157]. Current approaches rely largely on indirect clinical and microbiological indicators rather than real-time phenotypic assessment. For example, device-associated infections may suggest biofilm involvement, whereas reduced therapeutic response despite apparent susceptibility may indicate persistence or efflux-associated limitation [108,129,158].

Therapeutic selection within this framework aims to improve penetration, intracellular retention, or exposure duration through nanocarrier systems [26]. These approaches are intended to complement rather than replace conventional PK optimization.

Localized delivery increases antimicrobial availability within infected tissues, although therapeutic success remains dependent on adequate systemic pharmacokinetics and tissue distribution [69,115,116,117].

NP biodistribution and clearance may differ between infected and healthy tissues because inflammation, vascular changes, immune-cell recruitment, and local microenvironmental conditions can alter NP accumulation and retention at infection sites [69,91]. These differences may contribute to variable antimicrobial exposure across different infection settings [91,142]. In addition, uptake by the reticuloendothelial system can further influence systemic distribution and organ accumulation patterns [91].

Additional limitations include variability in host physiology, infection microenvironment, NP biodistribution, and the limited availability of clinically validated nanomedicine platforms [142,159].

Regulatory translation of antimicrobial nanomedicines is challenging because current approval pathways were originally developed for conventional drugs and do not fully account for the complex biological behavior of nanoscale systems [160,161]. Regulatory evaluation is further complicated by variability in NP size, shape, surface properties, and biological interactions [143]. Additional challenges include instability during large-scale manufacturing, batch-to-batch variation, uncertain biodistribution and organ accumulation, and limitations of some conventional toxicity assays due to NP interference [143,162]. Therefore, accurate physicochemical characterization, reproducible manufacturing, and comprehensive PK and safety assessment are essential for successful clinical development of nanotherapeutic platforms [143,160,161,162].

At present, phenotype-guided nanotherapy should be considered a translational framework rather than an established clinical algorithm because standardized phenotypic diagnostics and validated therapeutic thresholds remain unavailable.

## 6. Drug Repurposing Integrated with Nanotechnology

Within the phenotype-guided framework, drug repurposing expands therapeutic options beyond conventional antibiotic development. Increasing resistance in *A. baumannii*, including reduced susceptibility to last-resort agents, has intensified interest in alternative antimicrobial strategies [138,163].

Drug repurposing identifies new antimicrobial applications for clinically approved compounds with established pharmacological and safety profiles, thereby reducing development time and uncertainty compared with de novo antibiotic discovery [163,164,165]. Integration with nanotechnology further supports this strategy by improving drug localization, stability, and exposure within infection environments that restrict conventional antimicrobial activity [166,167].

Nanocarrier systems can alter biodistribution, prolong retention, and facilitate antimicrobial transport into structurally complex infection sites such as biofilms. These properties may enhance the activity of repurposed compounds under conditions associated with CRAB persistence and treatment failure [166,167].

### 6.1. Rationale for Drug Repurposing in Resistant Infections

Drug repurposing represents a practical strategy for addressing antimicrobial resistance through reassessment of approved compounds for antibacterial use. Because pharmacokinetic, toxicological, and safety characteristics are frequently already established, repurposed agents may progress more rapidly toward clinical evaluation [163,165].

This approach is particularly relevant in *A. baumannii*, where therapeutic options remain limited by extensive multidrug resistance. Screening studies have identified multiple clinically approved compounds with previously unrecognized antibacterial activity against MDR isolates [164,168].

Several repurposed compounds appear to act through mechanisms distinct from conventional antibiotics, including disruption of membrane-associated functions, interference with regulatory pathways, and modulation of bacterial adaptive signaling systems [125,165]. These alternative mechanisms may provide therapeutic value when conventional antibacterial targets become ineffective.

### 6.2. Repurposed Drug Classes with Antimicrobial Activity

Multiple classes of non-antibiotic compounds have demonstrated activity against *A. baumannii*, either alone or in combination with existing antimicrobials [125,169].

Antiparasitic agents and metabolic modulators represent one example. Niclosamide exhibits limited standalone activity but demonstrates synergistic effects with colistin against carbapenem-resistant strains [168]. Anti-inflammatory agents may also influence bacterial pathogenicity. Diclofenac has been reported to alter virulence-associated pathways, suggesting that some repurposed compounds can affect pathogenic behavior independently of direct bactericidal activity [170].

Additional compounds target membrane-associated processes. Farnesol disrupts biofilm structure and promotes bacterial killing without detectable resistance following repeated exposure under experimental conditions [171]. Fendiline has similarly been shown to interfere with lipoprotein trafficking and outer membrane assembly, increasing bacterial susceptibility [172].

Large-scale drug-screening studies further support the diversity of repurposing candidates. Compounds such as 5-fluorouracil and fluspirilene have demonstrated the ability to restore susceptibility to conventional antimicrobials, supporting their potential role as resistance-modifying adjuncts [164].

### 6.3. Nanoformulation to Enhance Targeting and Reduce Toxicity

Clinical application of repurposed compounds is often limited by inadequate antimicrobial potency, unfavorable pharmacokinetics, or insufficient penetration into infected tissues. Nanocarrier systems may help overcome these limitations through increased localization and prolonged antimicrobial retention, and protection from premature degradation [11].

Encapsulation can alter biodistribution and prolong local exposure, thereby maintaining antimicrobial activity against *A. baumannii* over extended periods [11,67,85]. Within biofilm-associated infections, NP systems promote exposure of protected bacterial subpopulations [173].

Combination nanocarrier systems capable of co-delivering repurposed agents with conventional antimicrobials may further enhance bactericidal activity and improve therapeutic response in resistant infections [174]. Surface modification and physicochemical tuning, including adjustment of charge, hydrophobicity, and particle composition, additionally influence cellular interaction, uptake, and biological behavior [87,88].

Despite these advantages, safety considerations remain critical. NP size, composition, surface reactivity, and dose-dependent oxidative stress responses can influence toxicity and immunogenicity [140,141]. Physiological ionic conditions and adsorption of serum proteins may alter NP surface characteristics, promote aggregation, and affect biodistribution, cellular uptake, and antimicrobial activity in vivo [69,91,175]. Consequently, optimization of nanocarrier design requires careful balancing of antimicrobial activity, biodistribution, and host compatibility [176].

## 7. Nano-Enabled Diagnostics and AI-Assisted Therapeutic Optimization

Advances in nano-enabled diagnostics and computational analysis support earlier identification of *A. baumannii* and characterization of resistance-associated features [157]. Compared with conventional culture-based workflows, these approaches shorten diagnostic turnaround time and facilitate earlier therapeutic decision-making. Integration of rapid diagnostics with computational analysis may support antimicrobial selection based on infection-specific characteristics [157,177].

### 7.1. Nano-Enabled Rapid Diagnostic Platforms

Nano-enabled diagnostic systems detect *A. baumannii* with high sensitivity using electrochemical and optical biosensing approaches [178]. These platforms can identify low-abundance targets while reducing analytical time compared with conventional microbiological methods.

Microfluidic and lab-on-chip technologies further support rapid point-of-care analysis by integrating sample processing and detection within miniaturized platforms [179]. Such systems may facilitate earlier intervention, particularly in critically ill patients requiring rapid therapeutic decisions.

Target-specific nano-biosensors developed for *A. baumannii* increase analytical sensitivity through signal amplification and label-free detection strategies [180,181,182]. In addition to bacterial detection, some platforms can identify resistance-associated features that may assist antimicrobial selection and therapeutic prioritization [157].

### 7.2. AI for Resistance Prediction and Therapy Optimization

AI-based analytical approaches are increasingly applied to diagnostic and clinical datasets to estimate resistance patterns in *A. baumannii*. Machine learning models trained on microbiological, genomic, and patient-derived data have demonstrated potential to predict antimicrobial susceptibility earlier than conventional testing workflows [177,183].

Clinical prediction models have also been used to identify patients at increased risk of *A. baumannii* infection, particularly in intensive care settings, thereby supporting earlier clinical management [184]. Genomic approaches further extend this capability. Deep learning models applied to sequencing data can identify resistance-associated determinants and infer susceptibility profiles without full reliance on culture-based methods [185].

AI-assisted screening has also contributed to antimicrobial discovery. Deep learning-guided analyses identified compounds with activity against *A. baumannii*, including abaucin, highlighting the potential of AI-assisted therapeutic development [186]. AI-based analysis may improve interpretation of complex diagnostic signals and reduce measurement variability [187].

However, most current applications are still investigational, and broader clinical use requires prospective validation, standardized datasets, and integration into routine clinical workflows. Differences in dataset composition, institutional sampling practices, and patient populations may introduce bias and reduce model performance across different clinical settings [183,184]. In addition, the limited interpretability of some deep learning models may complicate clinical decision-making and reduce confidence in AI-guided therapeutic recommendations [188].

### 7.3. Linking Diagnostic Outputs to Nanotherapeutic Design

Emerging strategies seek to integrate diagnostic outputs with therapeutic planning in *A. baumannii* infections. Resistance-associated features and clinical risk indicators generated through diagnostic and computational platforms may support selection of nanotherapeutic strategies and antimicrobial combinations [177,184].

Nano-enabled diagnostic systems can generate quantitative biosensing outputs reflecting bacterial burden and response-associated patterns. When integrated with computational analysis, these data may support therapeutic optimization, including selection of localized delivery systems or controlled-release formulations [189].

Although still investigational, this framework establishes a functional connection between diagnostic analysis and therapeutic design, supporting phenotype-guided intervention strategies [188,190].

**Figure 4:**
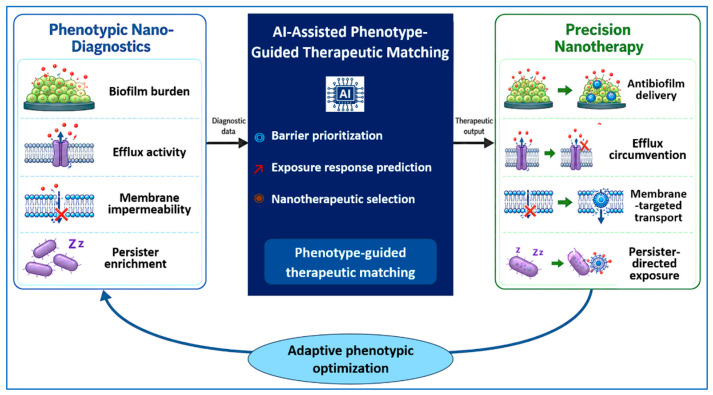
AI-assisted framework for phenotype-guided nanotherapy in CRAB infections. Phenotypic nano-diagnostics identify dominant barriers, including biofilm burden, efflux activity, membrane impermeability, and persister enrichment. These data are integrated within an AI-assisted decision-support system to prioritize barriers, predict exposure–response relationships, and guide nanotherapeutic selection. Adaptive phenotypic optimization supports continuous treatment refinement.

Nevertheless, clinical utility remains dependent on prospective validation, workflow standardization, and demonstration of reproducible benefit across diverse clinical settings [177,184]. Figure 4 illustrates an AI-assisted phenotype-guided framework linking nano-diagnostics, therapeutic matching, precision nanotherapy, and adaptive optimization.

## 8. Host-Directed and Microenvironment-Responsive Strategies

The integrated diagnostic–therapeutic framework described in Figure 4 can be extended beyond pathogen detection to include host-directed and microenvironment-responsive interventions influencing infection progression and therapeutic response. Conventional antimicrobials primarily target bacterial viability but often fail to address host factors and local tissue conditions contributing to persistence and treatment failure. This limitation has encouraged development of host-directed strategies aimed at modulating immune responses, regulating inflammation, and disrupting conditions that support bacterial survival [191].

In *A. baumannii* infections, altered immune activity, tissue damage, and disruption of resident microbial communities contribute to persistence and recurrence [192,193]. Nanotechnology may support these approaches through site-directed antimicrobial release and controlled therapeutic activation, and responsiveness to infection-associated signals [194,195].

### 8.1. Immune Modulation to Enhance Bacterial Clearance

Host-directed strategies aim to strengthen immune-mediated bacterial clearance while limiting excessive inflammatory injury [196]. Nanomaterials can interact with immune cells and influence antimicrobial immune responses. Macrophages are particularly important because they coordinate pathogen clearance and inflammatory signaling [197]. Several NP-based systems have been reported to promote macrophage polarization toward phenotypes associated with increased antimicrobial activity, potentially improving bacterial clearance [198,199].

Excessive inflammation, however, contributes to tissue injury and disease severity. Dysregulated cytokine responses have been associated with unfavorable outcomes in severe infections [196]. Engineered nanomaterials may help regulate inflammatory signaling pathways and support balanced immune activation while reducing collateral tissue damage [200].

Repeated administration of NP formulations may increase the risk of unintended immune activation or altered immune clearance [159,193]. NP composition, surface characteristics, and dosing frequency can influence immune recognition and affect long-term safety [193,201]. Therefore, careful evaluation of immunological compatibility and repeated-exposure safety is important during development of nanotherapeutic systems.

Some host-directed approaches also aim to improve intracellular bacterial clearance by promoting autophagy or phagolysosomal maturation [202,203]. However, evidence supporting these mechanisms in *A. baumannii* infections remains limited, and validation in CRAB-specific models remains limited.

### 8.2. Microenvironment-Responsive Nanotherapeutics

Infected tissues differ substantially from healthy microenvironments and commonly exhibit acidic pH, oxidative stress, and elevated enzymatic activity resulting from both bacterial metabolism and host inflammatory responses [113,204]. These characteristics provide physiologically relevant triggers for localized antimicrobial activation.

Stimuli-responsive nanotherapeutic systems are designed to respond to infection-associated conditions including pH variation, redox imbalance, oxidative stress, and enzymatic activity [114,205,206,207].

pH-sensitive carriers can enhance drug release within acidic biofilm-associated environments, thereby improving local antimicrobial availability [205]. ROS-responsive systems utilize elevated oxidative stress to activate antimicrobial effects while potentially reducing systemic exposure [208]. Enzyme-responsive systems similarly exploit bacterial enzymatic activity to trigger site-specific drug release [205].

These approaches may improve local drug concentration and reduce off-target exposure, particularly in *A. baumannii* biofilms where dense extracellular matrices and chemical gradients restrict antibiotic penetration [113]. Physiological ionic strength, serum proteins, and biofilm heterogeneity can also influence NP performance, serum protein interactions, and biofilm heterogeneity, which can alter stability, diffusion, and therapeutic performance under clinical conditions.

### 8.3. Microbiome-Oriented Interventions

The microbiome contributes importantly to colonization resistance against *A. baumannii*. Disruption of microbial communities, particularly in hospitalized or critically ill patients, increases susceptibility to colonization and infection [209,210]. The gastrointestinal tract represents an important reservoir, where microbial composition influences persistence, transmission, and infection risk [211,212].

Loss of microbial diversity may facilitate *A. baumannii* expansion by reducing microbial competition and altering host–microbe interactions [212]. In respiratory infections, reduced microbial diversity has been associated with increased microbial burden, dysregulated immune responses, and greater disease severity [213]. Conversely, stable microbial communities may limit pathogen persistence through nutrient competition, metabolite production, and host immune modulation [214].

Strategies aimed at restoring microbial balance have shown potential for reducing bacterial burden in experimental settings [215]. Selective nanotherapeutic delivery systems can support these approaches by minimizing disruption of commensal microbiota while maintaining antimicrobial effects against pathogens [216,217]. Preservation of microbiome stability may reduce recurrence risk and support post-treatment recovery [216,218].

The principal host-directed and microenvironment-responsive components integrated within this framework are summarized in Table 4.

## 9. Translational and Regulatory Considerations in Nanomedicine

Clinical implementation of phenotype-guided nanotherapeutic strategies depends on technologies that can operate reproducibly and safely in complex clinical settings. Despite promising antimicrobial applications, translation of nanotherapeutics from experimental systems to clinical practice remains limited by safety concerns, manufacturing challenges, and regulatory uncertainty.

NP-based platforms may improve drug localization and controlled release. However, their biological behavior, production requirements, and evaluation pathways differ substantially from those of conventional small-molecule drugs. These differences complicate clinical development and regulatory assessment [175,219].

### 9.1. NP Toxicity and Biodistribution

Safety remains a major consideration in nanomedicine development. NPs interact with biological systems through mechanisms influenced by size, surface charge, morphology, and composition, resulting in variable cellular uptake, immune recognition, and tissue distribution [142].

Several NP systems have demonstrated accumulation within organs such as the liver, spleen, and lungs, raising concerns regarding long-term retention and clearance [201]. Biodistribution is further influenced by administration route and interactions with biological fluids. Formation of a protein corona may alter NP targeting, cellular interaction, and circulation behavior [110].

At the cellular level, toxicity is frequently associated with oxidative stress and ROS generation, which can disrupt membranes, alter proteins, and damage nucleic acids [151,220]. Inflammatory and immunogenic responses may also occur because of unintended interactions with host immune pathways, particularly following repeated administration or prolonged exposure [173]. Rapid membrane disruption by some antimicrobial NPs may additionally promote endotoxin release from Gram-negative bacteria, potentially amplifying inflammatory responses during severe infection.

Predicting in vivo NP behavior remains challenging. Conventional PK models do not fully capture NP distribution patterns because systemic drug concentrations may not accurately reflect tissue-level exposure [69,91]. NP performance can also differ substantially between healthy and infected tissues owing to altered vascular permeability, inflammatory signaling, and local microenvironmental conditions. These factors complicate dose optimization and safety assessment, particularly in CRAB infections where therapeutic efficacy depends on achieving sufficient local exposure at infected sites.

### 9.2. Manufacturing, Scalability, and Reproducibility

Implementation of nanomedicines in clinical practice requires reproducible large-scale manufacturing. Production processes nevertheless remain technically demanding because small variations in particle size, morphology, surface characteristics, or composition can significantly influence biological behavior and therapeutic performance [144].

Scale-up from laboratory synthesis to industrial production presents additional challenges. Methods optimized under experimental conditions frequently lose consistency during large-scale fabrication, increasing batch-to-batch variability and reducing reproducibility [175]. These limitations directly affect regulatory evaluation and clinical reliability.

Manufacturing costs also remain substantially higher than those of conventional antimicrobials because NP synthesis, purification, and characterization require specialized infrastructure and analytical systems [175]. Multifunctional nanoplatforms may also introduce additional complexity through incorporation of targeting ligands, responsive materials, or combination payloads, complicating process standardization and quality control.

Standardized characterization protocols remain limited. The absence of harmonized methods for evaluating NP properties and biological performance complicates comparison across studies and weakens reproducibility [145,221,222]. Progress in this area will depend on scalable fabrication methods, robust quality-control frameworks, and standardized characterization criteria capable of supporting consistent clinical translation.

### 9.3. Regulatory Frameworks for Nanomedicine-Based Antimicrobials

Regulatory evaluation of nanomedicines remains complex because these systems combine characteristics of drugs, biomaterials, and delivery platforms. Existing regulatory pathways developed for conventional small molecules and biologics do not fully address NP-specific properties such as biodistribution, nano–bio interactions, long-term retention, and physicochemical instability [159,162].

Current toxicological frameworks may inadequately capture NP-associated effects, including aggregation behavior, surface reactivity, protein corona formation, and immune interactions [142]. Regulatory differences between agencies such as the FDA and EMA further complicate development by introducing variability in characterization requirements, safety evaluation, and product classification [162,223].

Manufacturing variability adds additional regulatory challenges because relatively minor formulation changes may alter pharmacokinetics, tissue distribution, therapeutic activity, or toxicity profiles [224]. Consequently, regulatory assessment increasingly requires detailed physicochemical characterization together with evaluation of biological performance and manufacturing consistency.

Although regulatory pathways for antimicrobial nanomedicines remain under development, ongoing efforts toward harmonized guidelines, nano-specific evaluation frameworks, and advanced analytical methodologies may improve future clinical translation and approval processes.

## 10. Future Perspectives: Toward Adaptive Phenotype-Guided Nanomedicine

The framework described in previous sections integrates diagnostics, computational analysis, and nanotherapeutic intervention to support phenotype-guided antimicrobial strategies. Future progress will depend on improving the ability of these systems to respond to changing infection conditions rather than relying solely on fixed therapeutic regimens [175,219]. Emerging developments in nanomedicine increasingly integrate real-time diagnostics, computational modeling, and responsive antimicrobial-delivery platforms to support phenotype-guided therapeutic decision-making.

### 10.1. Phenotype-Responsive Therapeutic Models

Antimicrobial therapy is progressively shifting from standardized treatment approaches toward strategies informed by infection-specific biological characteristics. This transition reflects recognition that bacterial populations exhibit pronounced physiological variability, including differences in metabolic activity, stress adaptation, and structural organization that influence antimicrobial susceptibility [118].

Within biofilms, nutrient and oxygen gradients generate functionally distinct bacterial subpopulations with variable responses to antimicrobial exposure [111]. Furthermore, subsets of bacteria may enter transient tolerant states, including persister phenotypes characterized by reduced metabolic activity and survival despite antimicrobial treatment [146]. These phenotypic states contribute to treatment failure and recurrence, particularly within heterogeneous infection microenvironments [112].

Integration of diagnostic information into therapeutic planning may help address this variability by enabling treatment adjustment according to current infection characteristics. Stimuli-responsive nanotherapeutic systems further support this concept through environmentally regulated drug release aligned with local infection conditions [225,226]. Real-time monitoring of phenotypic transitions remains difficult, and coexistence of multiple bacterial states within a single infection continues to complicate therapeutic optimization.

### 10.2. Integration of Diagnostics, Nanotechnology, and AI

Future antimicrobial strategies will increasingly integrate diagnostics, nanomaterials, and computational analysis. Combining nanoscale sensing systems with machine learning may improve pathogen detection and assist therapeutic selection through analysis of clinical and molecular datasets [227,228].

Integrated systems linking data acquisition, interpretation, and therapeutic response are under active investigation. AI-assisted models may support prediction of antimicrobial response and optimization of nanocarrier design based on biological and physicochemical parameters [227]. Therapeutic outcomes may also support iterative treatment refinement.

Despite this potential, implementation remains constrained by several challenges, including limited availability of standardized datasets, variability between clinical populations, and uncertainty regarding model interpretability and generalizability. Reliable clinical integration will therefore require prospective validation and robust analytical frameworks capable of supporting reproducible therapeutic recommendations [229].

### 10.3. Emerging Next-Generation Antimicrobial Platforms

Advances in nanomaterial engineering are enabling development of multifunctional antimicrobial systems integrating sensing, localized delivery, and therapeutic activity [230]. Stimuli-responsive nanomaterials can alter therapeutic activity in response to local environmental conditions such as pH, oxidative stress, or enzymatic activity, potentially improving site-specific antimicrobial delivery while reducing systemic exposure [231].

Bioinspired and programmable nanostructures may further improve selective interaction with microbial and host environments. Computational design approaches are also being explored to optimize nanomaterial properties for defined biological conditions [227].

Several limitations continue to limit real-world therapeutic application. Most supporting evidence for responsive nanotherapeutic systems is derived from in vitro and preclinical studies, whereas clinical validation remains limited [26]. Variability in infection site, host physiology, NP biodistribution, and local microenvironmental conditions may influence therapeutic performance [115,116].

NP stability under physiological ionic conditions, serum protein exposure, and polymicrobial environments may alter targeting efficiency and drug-release behavior during clinical application.

**Figure 5:**
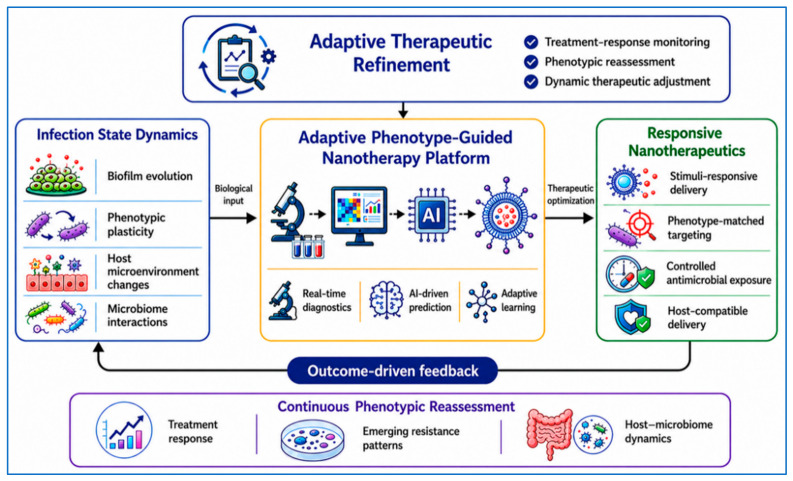
Adaptive phenotype-guided nanomedicine framework for CRAB infections. Dynamic infection-state factors, including biofilm evolution, phenotypic plasticity, host microenvironment changes, and microbiome interactions, inform an adaptive platform that integrates real-time diagnostics, AI-assisted prediction, and responsive nanotherapeutics. Outcome-driven feedback supports continuous phenotypic reassessment and therapeutic refinement, enabling adjustment of antimicrobial strategies as infection conditions evolve.

Consequently, phenotype-guided nanomedicine should currently be considered a conceptual and translational framework rather than an established therapeutic model. Clinical investigation will be necessary to determine its feasibility, reproducibility, safety, and impact on antimicrobial treatment outcomes. Figure 5 presents a conceptual adaptive framework that integrates infection-state dynamics, AI-assisted phenotypic analysis, responsive nanotherapeutic strategies, and continuous phenotypic reassessment to support future phenotype-guided antimicrobial intervention.

## 11. Conclusions

CRAB remains a major clinical challenge because antimicrobial failure is shaped not only by genetic resistance but also by dynamic phenotypic states that influence drug exposure and bacterial survival during infection. This review presents a phenotype-guided framework linking functional resistance-associated states with nanotherapeutic strategies designed to improve antimicrobial delivery, retention, and activity within structurally and physiologically complex infection settings. By aligning therapeutic intervention with dominant biological constraints such as biofilm formation, efflux activity, membrane impermeability, and persistence, nanotherapeutic systems support more targeted antimicrobial activity than conventional empirical approaches. Integration of diagnostic platforms, computational analysis, and responsive delivery systems further highlights the potential for more individualized therapeutic decision-making. Despite these advances, substantial translational barriers remain, including incomplete phenotypic characterization, variability in infection microenvironments, uncertainties regarding NP biodistribution and safety, manufacturing complexity, and evolving regulatory frameworks. In addition, most supporting evidence remains derived from experimental and preclinical studies, while few nanotherapeutic platforms for CRAB have reached clinical validation. Future progress will depend on development of rapid phenotypic diagnostics, standardized evaluation frameworks, scalable nanomedicine manufacturing strategies, and robust clinical validation under real infection conditions. Collectively, these advances support development of phenotype-guided antimicrobial strategies better aligned with the biological conditions governing CRAB infections.

## Figures and Tables

**Table 1:** Functional resistance phenotypes in CRAB and their impact on antimicrobial performance.

Phenotypic Trait	Functional Basis	Effect on Antibiotic Activity	Clinical Relevance	Representative Nanotherapeutic Strategy	Refs.
Biofilm-associated growth	Extracellular matrix formation with diffusion limitation and microenvironmental gradients	Reduced penetration and diminished activity in metabolically inactive subpopulations	Persistence in device-associated and chronic infections	Liposomal and polymeric antibiofilm nanocarriers	[38,39,40,42]
Efflux-mediated extrusion	RND transport systems (e.g., AdeABC) driven by proton motive force	Decreased intracellular antibiotic concentration across multiple drug classes	Multidrug resistance and reduced treatment efficacy	Membrane-disruptive or efflux-modulating NPs	[33,34,48,51]
Outer membrane adaptation	Altered porins (e.g., CarO) and reduced permeability of the outer membrane	Limited antibiotic entry into periplasmic space	Intrinsic resistance and reduced susceptibility to carbapenems	Cationic and membrane-interacting nanocarriers	[31,57,58,59]
Persister cell formation	Reversible metabolic dormancy under stress conditions	Survival during exposure to bactericidal antibiotics	Relapse and incomplete infection clearance	Sustained-release and stimuli-responsive nanocarriers	[44,60]

**Table 2:** Comparative characteristics of major nanotherapeutic platforms investigated against CRAB.

Nanotherapeutic Platform	Major Advantages	Major Limitations	Representative Antimicrobial Characteristics	Refs.
Lipid-based nanocarriers	Increase membrane interaction and drug delivery	Physical instability and possible drug leakage	Biofilm penetration and intracellular antimicrobial transport	[85,93,94]
Polymeric NPs	Provide sustained antimicrobial release	Complex formulation and release variability	Controlled delivery and biofilm disruption	[83,90,97]
Metallic NPs	Broad antimicrobial activity through multiple targets	Cytotoxicity and oxidative stress concerns	Membrane damage and antibiotic synergy	[52,101,104]
Stimuli-responsive nanoplatforms	Trigger site-specific antimicrobial release	Limited clinical validation	pH-, ROS-, and enzyme-responsive delivery	[24,113,114]

**Table 3:** Phenotype-Oriented Nanotherapeutic Strategies for CRAB.

Phenotypic Constraint	Constraint Type	Primary Exposure Limitation	Nanotherapeutic Strategy	Mechanistic Action	Therapeutic Outcome	Limitation	Refs.
Biofilm architecture	Diffusion-limited	Reduced penetration within structured matrix	Matrix-interactive nanosystems	Facilitate matrix interaction; promote intrabiofilm transport	Improved distribution across biofilm layers	Limited penetration in dense matrix environments	[17,110,120]
Efflux overactivity	Transport-dominated	Reduced intracellular retention	Efflux-modulating nanomaterials	Interfere with efflux activity; enhance intracellular persistence	Increased intracellular drug levels	Variable inhibition due to regulatory adaptation	[33,48,129,130]
Membrane impermeability	Entry-limited	Restricted cellular uptake	Membrane-interacting nanocarriers	Promote membrane interaction; support translocation	Increased intracellular delivery	Potential toxicity linked to membrane disruption	[56,88,136,140]
Persister formation	Tolerance-associated	Reduced antibiotic success despite presence	Metabolism-independent nanotherapeutics	Induce membrane damage or oxidative stress; support prolonged exposure	Reduced persistence and recurrence	Effect depends on sufficient exposure duration	[43,140,146]
Mixed phenotypes	Multi-constraint	Combined exposure limitations	Multifunctional nanoplatforms	Integrate multiple mechanisms targeting entry, retention, and distribution	Broader antimicrobial activity	Efficacy depends on dominant constraint	[118,153,154]

**Table 4:** Host-Directed and Microenvironment-Responsive Components within the Phenotype-Guided Framework.

Component	System Position	Trigger/Input	Action	Outcome
Immune modulation	Input (host-derived signals)	Immune activity, cytokine patterns	Modulation of immune activity and intracellular defense	Increased bacterial clearance with controlled inflammation
pH-responsive systems	Execution (local activation)	Acidic conditions in infected tissue and biofilms	Localized drug release at infection sites	Higher drug concentration with reduced systemic exposure
ROS-responsive systems	Execution (local activation)	Elevated oxidative stress	Activation of antimicrobial activity under inflammatory conditions	Reinforced antibacterial effect in stressed environments
Enzyme-responsive delivery	Execution (local activation)	Bacterial enzymatic activity	Site-specific drug release triggered by pathogen presence	Increased targeting accuracy and reduced off-target effects
Microbiome-oriented strategies	Feedback (ecosystem response)	Changes in microbial composition	Restoration of microbial balance and colonization resistance	Reduced pathogen persistence and recurrence
Microbiome-sparing delivery	Cross-link (execution and feedback)	Selective targeting of pathogenic bacteria	Antimicrobial action with limited disruption of commensals	Preservation of microbiome stability and recovery

## Data Availability

No new data were created or analyzed in this study. Data sharing is not applicable to this article.
